# Planned Yellow Fever Primary Vaccination Is Safe and Immunogenic in Patients With Autoimmune Diseases: A Prospective Non-interventional Study

**DOI:** 10.3389/fimmu.2020.01382

**Published:** 2020-07-17

**Authors:** Valéria Valim, Ketty Lysie Libardi Lira Machado, Samira Tatiyama Miyamoto, Arthur Dalmaso Pinto, Priscila Costa Martins Rocha, Erica Vieira Serrano, Valquiria Garcia Dinis, Sônia Alves Gouvêa, João Gabriel Fragoso Dias, Ana Carolina Campi-Azevedo, Andréa Teixeira-Carvalho, Vanessa Peruhype-Magalhães, Ismael Artur da Costa-Rocha, Sheila Maria Barbosa de Lima, Emily Hime Miranda, Gisela Freitas Trindade, Maria de Lourdes de Sousa Maia, Maria Bernadete Renoldi de Oliveira Gavi, Lidia Balarini da Silva, Ruben Horst Duque, Ana Paula Espíndula Gianordoli, Thays Zanon Casagrande, Karine Gadioli Oliveira, Bruna Costa da Mata Moura, Fernanda Nicole-Batista, Luiza Correa Rodrigues, Thalles Brandão Clemente, Enan Sales Magalhães, Maria de Fatima Bissoli, Maria da Penha Gomes Gouvea, Lauro Ferreira da Silva Pinto-Neto, Carolina Zorzanelli Costa, Raquel Altoé Giovelli, Leticia Resende Brandão, Elizandra Tomazela Laurenti Polito, Ingrid de Oliveira Koehlert, Brunela Passos Borjaille, Daniela Bergamim Pereira, Laiza Hombre Dias, Daniela Linhares Merlo, Luiz Fellipe Favoreto Genelhu, Flavia Zon Pretti, Maryella dos Santos Giacomin, Ana Paula Neves Burian, Francieli Fontana Sutile Tardetti Fantinato, Gecilmara Salviato Pileggi, Lícia Maria Henrique da Mota, Olindo Assis Martins-Filho

**Affiliations:** ^1^Divisão de Reumatologia do Hospital Universitário Cassiano Antônio de Moraes, Universidade Federal do Espírito Santo (UFES), Vitória, Brazil; ^2^Escola de Ciências da Saúde da Santa Casa de Misericórdia, Vitória, Brazil; ^3^Instituto René Rachou, Fundação Oswaldo Cruz (FIOCRUZ-Minas), Belo Horizonte, Brazil; ^4^Instituto de Tecnologia em Imunobiológicos (Bio-Manguinhos), Fundação Oswaldo Cruz (FIOCRUZ), Rio de Janeiro, Brazil; ^5^Sociedade de Reumatologia do Espírito Santo (SORES), Vitória, Brazil; ^6^Centro de Referências para Imunobiológicos Especiais (CRIE) da Secretaria de Saúde do Estado do Espírito Santo, Vitória, Brazil; ^7^Departamento de Vigilância das Doenças Transmissíveis, Secretaria de Vigilância em Saúde, Ministério da Saúde, Brasília, Brazil; ^8^Faculdade de Ciências da Saúde de Barretos—FACISB, Barretos, Brazil; ^9^Divisão de Reumatologia do Hospital Universitário de Brasília, Faculdade de Medicina, Universidade de Brasília, Brasília, Brazil

**Keywords:** yellow fever vaccine, autoimmune diseases, viremia, seroconversion, pharmacokinetics

## Abstract

Yellow Fever (YF) vaccination is suggested to induce a large number of adverse events (AE) and suboptimal responses in patients with autoimmune diseases (AID); however, there have been no studies on 17DD-YF primary vaccination performance in patients with AID. This prospective non-interventional study conducted between March and July, 2017 assessed the safety and immunogenicity of planned 17DD-YF primary vaccination in patients with AID. Adult patients with AID (both sexes) were enrolled, along with healthy controls, at a single hospital (Vitória, Brazil). Included patients were referred for planned vaccination by a rheumatologist; in remission, or with low disease activity; and had low level immunosuppression or the attending physician advised interruption of immunosuppression for safety reasons. The occurrence of AE, neutralizing antibody kinetics, seropositivity rates, and 17DD-YF viremia were evaluated at various time points (day 0 (D0), D3, D4, D5, D6, D14, and D28). Individuals evaluated (*n* = 278), including patients with rheumatoid arthritis (RA; 79), spondyloarthritis (SpA; 59), systemic sclerosis (8), systemic lupus erythematosus (SLE; 27), primary Sjögren's syndrome (SS; 54), and healthy controls (HC; 51). Only mild AE were reported. The frequency of local and systemic AE in patients with AID and HC did not differ significantly (8 vs. 10% and 21 vs. 32%; *p* = 1.00 and 0.18, respectively). Patients with AID presented late seroconversion profiles according to kinetic timelines of the plaque reduction neutralization test (PRNT). PRNT-determined virus titers (copies/mL) [181 (95% confidence interval (CI), 144–228) vs. 440 (95% CI, 291–665), *p* = 0.004] and seropositivity rate (78 vs. 96%, *p* = 0.01) were lower in patients with AID after 28 days, particularly those with SpA (73%) and SLE (73%), relative to HC. The YF viremia peak (RNAnemia) was 5–6 days after vaccination in all groups. In conclusion, consistent seroconversion rates were observed in patients with AID and our findings support that planned 17DD-YF primary vaccination is safe and immunogenic in patients with AID.

## Introduction

The 17DD-Yellow Fever (YF) vaccine induces safe and effective protective immunity in healthy individuals, resulting from robust humoral and cellular immune responses (1–3); however, it has been proposed that immune-compromised individuals mount suboptimal immunologic responses after vaccination (4–6). Moreover, some studies have pointed to a high prevalence of severe adverse post-vaccination events in patients with autoimmune diseases (AID), particularly systemic lupus erythematosus (SLE) and those receiving systemic corticosteroid therapy (7–10). Studies assessing the safety, effectiveness, and immunogenicity of YF vaccination in immune-compromised patients, particularly those with AID, remain scarce (4).

There is still no antiviral treatment for YF, therefore prevention actions such as mosquito control, protection from mosquito bite and vaccination are extremely necessary. A live attenuated vaccine strain 17D was developed in 1937. Two substrains are used in the vaccine today, substrains 17D-204 (Sanofi- Pasteur) and 17DD (Fiocruz), which are at passages 235–240 and 287–289, respectively, from wild-type Asibi virus (11).

The vaccine produces high level of protection that occurs in 90% of vaccines within 10 days and in nearly 100%, in 4 weeks. Immunity after a single dose is long lasting and may provide protection for life (12). The World Health Organization (WHO) recommends a single dose immunization for travelers to endemic area. However, protective cellular and humoral immunity wanes over time in some individuals (13).

YF vaccination is generally well-tolerated, adverse events are reported in only 43 per 100,000 doses and most cases are mild. “Vaccine-Associated Viscerotrophic Disease” (YEL-AVD) and “Vaccine-Associated Neurological Disease” (YEL-AND) are severe and rare adverse events, reported only in primary vaccinees, and especially in children, elderly and history of thymus disease (11, 14).

In December 2016, a YF outbreak occurred in Brazil that extended to several Eastern states, including areas not traditionally considered at risk and where, therefore, YF vaccination was not recommended to the resident populations, or travelers to those specific locations, until the outbreak. YF is a severe infectious disease and vaccination is the most important way to protect from this condition, which has high mortality rates. Soon after the first cases were reported in 2017, the Brazilian Government decided to conduct an extensive Brazilian YF vaccination campaign. Immunization was free and offered by many public services in the affected zones; consequently, numerous patients with AID were inadvertently vaccinated or remained unvaccinated and susceptible, and at risk of YF infection and its severe outcome.

Live attenuated vaccines should be used with caution in populations with AID because of the risk of adverse events (AE). The majority of guidelines generally recommend avoiding live vaccines for immunosuppressed individuals (15). The decision to be vaccinated must consider both the risks of exposure and possibility of death from YF, and the risks of complications caused by the vaccine (16). Recently, the Brazilian Society of Rheumatology, Dermatology, Bowel Inflammatory Disease have published recommendations about YF vaccination in patients with chronic immune-mediated inflammatory diseases living or traveling to YF endemic areas (17). Faced with absence of prospective studies in AID, it is necessary to establish medical evaluation criteria to allow or prohibit vaccination.

To date, there have been no studies investigating the response to, and safety of, planned 17DD-YF primary vaccination in patients with AID patients. Therefore, any effort to generate scientific evidence will contribute to development of appropriate recommendations regarding vaccination. The aims of this study were to evaluate the occurrence of AE, seroconversion rates, kinetics of neutralizing antibody production, and vaccine viremia after 17DD-YF primary vaccination of patients with AID.

## Materials and Methods

### Study Design

This was a prospective non-interventional study, carried out between March 2017 and July 2017 in Vitória, Espírito Santo, Brazil. All participants received the 17DD-YF primary vaccination (Bio-Manguinhos-FIOCRUZ) during the 2017 Brazilian YF vaccination campaign, coordinated by the State Government. This study is registered in the Registro Brasileiro de Ensaios Clínicos (Brazilian Registry of Clinical Trials, UTN# U1111-1217-6672).

Individuals of both sexes, aged from 18 to 88 years, with the following AID diagnoses: rheumatoid arthritis (RA), spondyloarthritis (SpA), systemic sclerosis (SSC), systemic lupus erythematosus (SLE), Sjögren's syndrome (SS), and healthy controls (HC), were enrolled in the study. Patients with AID were attended in the Rheumatology Outpatient Unit of Hospital Universitário Cassiano Antônio Moraes/EBSERH at Universidade Federal do Espírito Santo (HUCAM-UFES/EBSERH), where the risks and safety of the YF vaccine were evaluated. The HC group consisted of individuals who attended the routine vaccination unit at HUCAM. All those did not have AID and did not meet the exclusion criteria.

The study was submitted and approved by the ethical committee of HUCAM-UFES/EBSERH (C.A.A.E 65910317.0.0000.5071, approval #2.411.738/2017). Informed consent was obtained from all participants.

### Inclusion/Exclusion Criteria

The inclusion criteria for both groups comprised: individuals > 18 years, able to understand and read the consent form, or have a legal representative to read it, and had never received YF vaccination. Moreover, in the AID group, each patient fulfilled international classification criteria for AID, according to the American College of Rheumatology and/or European League Against Rheumatism international classification criteria for RA, SpA, SSC, SLE, and SS (18–23). All patients were advised by a rheumatologist to undergo planned YF vaccination when in remission or had low disease activity; and, when using immunosuppressant or biological therapy were advised that it was safe to interrupt this by their physician. The interval between withdrawal of therapy and YF vaccination was that specified in the Brazilian Recommendations for YF vaccination in patients with AID (17), as follows: interval > 3 months for immunosuppressive oral therapy, > 5.5 half-lives for any biological therapy, and ≥ 6 months for rituximab (Table 1) (6, 17, 24, 25).

**Table 1:** Minimum period of time recommended between withdrawal of therapy and 17DD-YF vaccination for patients with AID, according to Brazilian recommendations *^a^*.

**Drug**	**Interval between withdrawal and vaccination**
Prednisone > 20 mg/day or pulse methylprednisolone	≥ 1 month
Hydroxychloroquine, sulfasalazine, acitretin, methotrexate ≤ 20 mg/week, leflunomide 20 mg/day	Consider vaccination without interval
Methotrexate > 20 mg/week	≥ 1 month
Azathioprine, mycophenolate, cyclosporine, tacrolimus, cyclophosphamide	≥ 3 months
Tofacitinib	≥ 2 weeks
Anti-cytokines and co-stimulation inhibitor	4–5 half-lives^b^
B-lymphocyte depletors	6–12 months

a *The medical criteria to conduct the drug elimination protocol before vaccination are indicated (13)*.

b *Based on pharmacological half-life, except B-lymphocyte depletors*.

Exclusion criteria comprised: patients who had not been advised by a rheumatologist to receive the vaccine; did not agree to participate; immunosuppressed by other causes (HIV carriers with CD4 count <200 cells/mm^3^ or lymphocytes <500 cells/mm^3^); low IgG or IgM levels; organ transplantation history; primary immunodeficiency; neoplasia; previous history of thymus diseases (myasthenia gravis, thymoma, thymus absence, or surgical removal); high disease activity index; receiving high levels of immunosuppressive treatment with cyclophosphamide, mycophenolate mofetil, tacrolimus, cyclosporine, sirolimus, azathioprine > 2 mg/kg/day, prednisone ≥ 20 mg/day, methotrexate > 20 mg/week, or any immunobiological drug (17, 24, 25); and received another vaccine simultaneously or at an interval <30 days. Individuals previously vaccinated against YF, according to their medical records, and those with seropositive results for anti-YF antibody by plaque reduction neutralization test (PRNT ≥ 1:50 at baseline) were also excluded.

### AID-Related Clinical Records

Baseline demographic data included AID classification criteria (18–23), disease duration (years), AID disease activity score (26–30), and current use of synthetic and biological disease-modifying anti-rheumatic drugs (DMARDs). Twenty-eight days after 17DD-YF primary vaccination, AID related symptoms, AID disease activity score, and AID-related symptoms were reassessed. All data collected were obtained by medical/nurse interview and current medical reports/prescriptions.

### Safety Assessment

At baseline, all patients were given a diary that contained information about all YF vaccine-related AE and were instructed to record any new symptom that presented up to 30 days after YF vaccination. They also received an appointment for a follow-up visit (D28) and examinations (as specified below). Unscheduled visits were permitted whether any new symptoms presented after vaccination. Symptoms recorded in the diary were confirmed during nurse/medical visits (unscheduled visits and/or D28 scheduled return visit). AE events were stratified by extent and severity, according to the WHO classification (31). Local AE were defined as any symptom, including pain, pruritus, hyperemia, edema, or node at the application site. Systemic AE were defined as any symptom including fever, headache, myalgia, arthralgia, weakness, tremor, urticaria, angioedema, anaphylactic reaction, jaundice, and peripheral edema. Severe AE were defined as YF vaccine-associated neurotropic disease, YF vaccine-associated viscerotopic disease, or complications that resulted in hospitalization or death. Mild AE were any other AE that did not meet the criteria for severe AE. For all AE, participants were actively asked about the symptoms and answered “yes” or “no.”

### Blood Samples

Blood samples were collected from each participant at baseline (day 0; D0) and at three subsequent scheduled time points: (i) [D3, D6, D28]; (ii) [D4, D7, D28]; or (iii) [D5, D14, D28]. Serum samples were obtained from 20 mL of whole blood collected in vacuum tubes without anticoagulant. Serum aliquots were stored at −80°C until processing for detection of neutralizing antibodies and viremia analysis.

### Analysis of YF Neutralizing Antibodies and Viremia Levels

YF vaccine immunogenicity was evaluated in serum samples by assessment of anti-YF neutralizing antibody levels using PRNT, which is the gold-standard method (32). The results are expressed as the reciprocal of serum dilution. Values above serum dilution 1:50 were considered positive. Viremia levels (YF viral RNAnemia) were quantified in serum samples by qRT-PCR assay, according to Martins et al. (33). The results are expressed as copies/mL. Samples were processed in Laboratório de Tecnologia Virológica, Bio-Manguinhos (LATEV, FIOCRUZ-RJ, Brazil).

### Statistical Analysis

Descriptive statistical analysis was conducted using Prism 5.03 software (GraphPad Software, San Diego, USA). A chi-square test was used to compare the occurrence of AE and PRNT seropositivity rates amongst groups. Comparative analysis of PRNT titers between the HC and AID groups was performed by Mann-Whitney test. Multiple comparisons of PRNT titers and viremia levels amongst HC and AID subgroups were carried out using the Kruskal-Wallis test, followed by Dunn's post-test for sequential pair-wise comparisons. In all cases, *p* < 0.05 were considered statistically significant.

## Results

**Figure 1:**
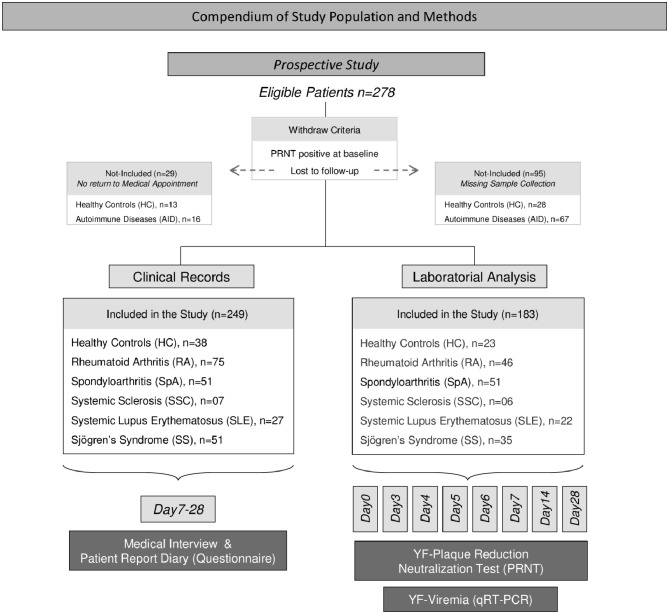
Overview of the study population and methods. This was a prospective non-interventional study carried out between March and July 2017 in Vitória, Espírito Santo, Brazil. The study enrolled 278 individuals of both sexes, ranging from 18 to 88 years old. Individuals seropositive for anti-YF antibody (PRNT ≥ 1:50 at baseline) or those lost to follow-up were withdrawn from the study after being included. A total of 249 volunteers completed the clinical records, comprising: 75 with rheumatoid arthritis (RA), 51 with spondyloarthritis (SpA), 7 with systemic sclerosis (SSC), 27 with systemic lupus erythematosus (SLE), 51 with Sjögren's syndrome (SS), and 38 healthy controls (HC). Clinical records were obtained by medical/nurse interview, from patient report diary or previous medical reports, prescriptions, and records, from D7 to D28 after 17DD primary vaccination. For laboratory analyses, a group of 183 volunteers (RA = 46, SpA = 51, SSC = 6, SLE = 22, SS = 35, HC = 23) agreed to have blood samples collected at baseline (D0) and subsequent time-points, including: D3, D4, D5, D6, D7, D14, and D28. Laboratory analyses included YF-plaque reduction neutralization test (PRNT) and YF viremia (RNAnemia) analysis by qRT-PCR.

In total, 278 individuals were included in the study: RA (*n* = 79), SpA (*n* = 59), SSc (*n* = 8), SLE (*n* = 27), SS (*n* = 54), and HC (*n* = 51). The mean [standard deviation; SD] age of participants in the AID group was 51 (14) years and 71.8% were women. In the HC group, mean [SD] age was 56 (15) years and 56.9% were women. At baseline, all individuals were in remission, or had low disease activity, and most were under low level immunosuppression (prednisone ≤ 20 mg/day; methotrexate ≤ 20 mg/week, azathioprine ≤ 2 mg/kg/day; leflunomide, sulfasalazine, or hydroxychloroquine). Few were undergoing strong immunosuppression (16.75% of RA and 49% of SpA were receiving biological therapy; 11.11% were receiving cyclophosphamide in the SLE group; 14.81% were on high doses of prednisone or methylprednisolone; and 29.63% were receiving azathioprine). In these patients with very stable disease, biological therapy and immunosuppressive therapy were discontinued before vaccination, according to Brazilian recommendations (17). Detailed clinical features of participants are provided in Table 2. The number of participants is shown in Figure 1.

**Table 2:** Baseline demographic, clinical, and therapeutic characteristics.

**Features**	**HC (*n =* 51)**	**AID (*n =* 227)**	**RA (*n =* 79)**	**SpA (*n =* 59)**	**SSC (*n =* 8)**	**SLE (*n =* 27)**	**SS (*n =* 54)**
Women, %	57	72	82	52	75	100	98
Age, mean (SD), years	56 (15)	51 (14)	55 (13)	47 (11)	59 (7)	45 (16)	54 (14)
PRED ≤ 20 mg/d, %	–	12.1	16.3	1.8	12.5	25.9	9.3
MTX, %	–	28.8	36.3	31.6	12.5	11.1	24.1
LFN, %	–	9.4	18.3	7.0	0	0	3.7
HCQ, %	–	17.1	13.8	1.8	0	44.4	25.9
SSA, %	–	4.9	2.5	15.8	0	0	0
AZA, %	–	5.9	0	0	12.5	29.6	7.4
MMF, %	–	1.3	0	0	0	7.4	1.9
CSA, %	–	0.4	1.3	0	0	0	0
CFM, %	–	2.3	1.3	0	12.5	11.1	0
PRED > 20 mg/d, %	–	2.7	0	0	0	14.8	3.7
Biological Therapy^a^, %	–	18.4	16.8	49.1	0	0	0
Disease Activity, mean (SD)	–	–	DAS 28 2.99 ± 0.9	BASDAI 1.92 ± 2.1	–	SLEDAI 1.08 ± 1.5	ESSDAI 1.89 ± 3.2

*HC, healthy controls; AID, autoimmune disease patients; RA, rheumatoid arthritis; SpA, spondyloarthritis; SSC, systemic sclerosis; SLE, systemic lupus erythematosus; SS, primary Sjögren's syndrome; SD, standard deviation; PRED, prednisone; MTX, methotrexate; LFN, leflunomide; HCQ, hydroxychloroquine; SSA, sulfasalazine; AZA, azathioprine; MMF, mycophenolate; CSA, cyclosporine; CFM, cyclophosphamide; DAS 28, disease activity score; BASDAI, bath ankylosing spondylitis disease activity index; SLEDAI, systemic lupus erythematosus disease activity index; ESSDAI, EULAR Sjögren's syndrome disease activity index*.

a *biological therapy included: adalimumab, etanercept, infliximab, abatacept and rituximab*.

### Safety of the 17DD-YF Vaccine

In the present study the occurrence of adverse events in both groups, HC and AID patients, was monitored by active surveillance based on the weekly medical visit and patient diary reports up to 28 days after 17DD-YF primary vaccination. A total of 249 clinical records, including 211 from patients with AID and 38 from HC, were obtained by interview and patient diary reports. Twenty-nine individuals were lost during follow-up. The frequency of lost during follow-up was around 25% in HC and 7% in AID. The frequencies of local and systemic AE observed after 17DD-YF primary vaccination are provided in Table 3. Only mild AE were reported. The analysis of local and systemic AE did not reveal significant differences in AID patients relative to HC (8 vs. 10% and 21 vs. 32%; *p* = 1.00 and 0.18, respectively).

**Table 3:** Adverse events in patients with autoimmune diseases after 17DD-YF primary vaccination.

**Groups**		**Adverse events (AE)**	
	**Local^a^, % (n)**	***p*-value**	**Systemic^b^, % (n)**	***p*-value**
**HC** (*n =* 38)	8 (3)	–	21 (8)	–
**AID** (*n =* 211)	21 (44)	1.00	32 (7)	0.18
**RA** (*n =* 75)	9 (7)	1.00	31 (23)	0.37
**SpA** (*n =* 51)	4 (2)	0.65	26 (13)	0.80
**SSC** (*n =* 07)	14 (1)	0.50	57 (4)	0.07
**SLE** (*n =* 27)	4 (1)	0.63	30 (8)	0.56
**SS** (*n =* 51)	2 (1)	0.14	39 (20)	010

*Comparative analysis between HC and AID or AID subgroups were carried out by χ2 test. p-values are reported for comparisons to HC*.

a *local AE included: pain, pruritus, hyperemia, edema, or node at the application site*;

b *systemic AE included: fever, headache, myalgia, arthralgia, weakness, tremor, urticaria, angioedema, anaphylactic reaction, jaundice, peripheral edema. HC, healthy controls; AID, autoimmune disease patients; RA, rheumatoid arthritis; SpA, spondyloarthritis; SSC, systemic sclerosis; SLE, systemic lupus erythematosus; SS, primary Sjögren's syndrome*.

### Immunogenicity of the 17DD-YF Vaccine

**Figure 2:**
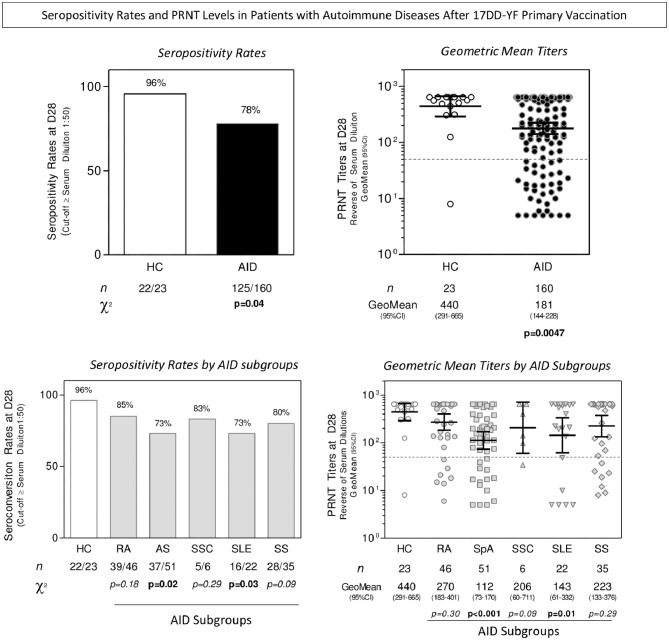
Seropositivity rates and PRNT levels after 17DD-YF primary vaccination in patients with AID. Levels of 17DD-YF specific neutralizing antibodies were detected by micro-PRNT, as previously described by Simões et al. (25). Seropositivity rates were determined with serum dilution ≥ 1:50 as the cut-off criterion for PRNT positivity (dashed line). Data are presented as bar charts of proportion of seropositive results at D28 according to the cut-off of 1:50 expressed in reverse of serum dilution for HC (□), AID (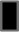), and AID subgroups (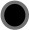). The chi-square test was employed for comparative analysis of PRNT seropositivity rates amongst groups. The PRNT levels at D28 are expressed as geometric mean titer and 95% CI of reverse serum dilution, presented in scatter plots for HC (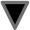), AID (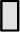), RA (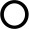), SpA (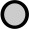), SSC (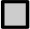), SLE (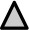), and SS (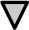). The cut-off of seropositivity is indicated by the dashed line (PRNT ≥ 1:50). Comparative analysis of PRNT titers between HC and AID groups was performed by Mann-Whitney test. Multiple comparisons of PRNT titers amongst HC and AID subgroups were conducted by Kruskal-Wallis test followed by Dunn's post-test for sequential pair-wise comparisons. In all cases, a threshold *p* < 0.05 was considered statistically significant. The number of samples tested for HC, AID, and AID subgroups is provided in the figure. HC, healthy contros; AID, autoimmune patients; RA, rheumatoid arthritis; SpA, spondyloarthritis; SSC, systemic sclerosis; SLE, systemic lupus erythematosus; SS, primary Sjögren's syndrome.

Seropositivity rates and PRNT levels in patients with AID at D28 after 17DD-YF primary vaccination are presented in Figure 2. Seropositivity rates (PRNT ≥ 1:50) were lower in patients with AID than HC (78 vs. 96%, *p* = 0.01). Comparative analysis of seropositivity rates among HC and AID subgroups demonstrated similar results for RA, SSC, and SS; however, lower seropositivity rates were observed in SpA (73%, *p* = 0.02) and SLE (73%, *p* = 0.03) relative to HC.

Analysis of PRNT levels demonstrated lower geometric mean titers in patients with AID relative to HC (181, 95% confidence interval (CI) 144–228 vs. 440, 95% CI 291–665; *p* = 0.004). Further comparative analysis among AID subgroups did not demonstrated significant differences between RA, SSC, and SS as compared to HC. Conversely, lower geometric mean titers were observed in patients with SpA (112, 95% CI 73–170; *p* < 0.001) and SLE (143, 95% CI 61–332; *p* = 0.01) relative to HC.

### Kinetic Timeline of PRNT Levels

**Figure 3:**
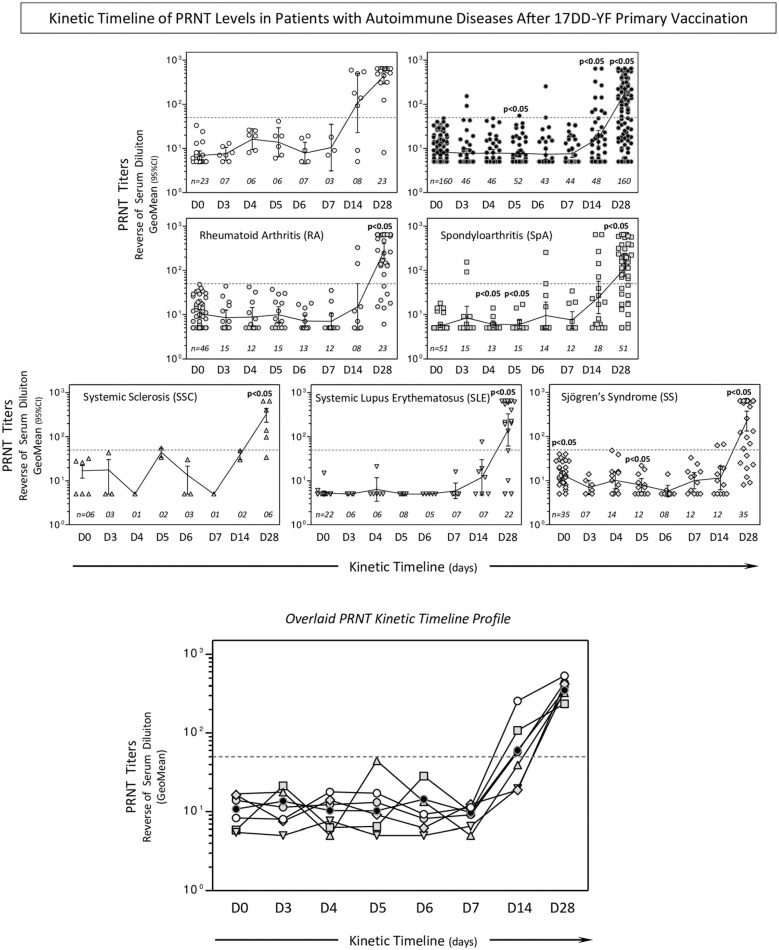
Kinetic timeline of PRNT levels in patients with AID after 17DD-YF primary vaccination. Levels of 17DD-YF specific neutralizing antibodies were detected by micro-PRNT, as previously described by Simões et al. (25). Data are presented as a scatter plot over a column chart of PRNT titers, expressed as the reverse of the serum dilution and 95% CI of reverse serum dilution (HC,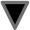; AD,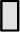; RA,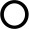; SpA,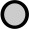; SSC,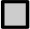; SLE,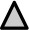; and SS,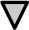) at baseline (day 0; D0) and over time after primary vaccination (D3, D4, D5, D6, D7, D14, and D28). The cut-off point (PRNT ≥ 1:50) is represented as a dashed line. Comparative analysis of PRNT titers at each time point (HC vs. AID or AID subgroups) was performed by Mann-Whitney test. A threshold *p* < 0.05 was considered statistically significant. Overlaid kinetic timeline profile of PRNT is also provided in the figure. The number of samples tested for HC, AID, and AID subgroups is provided in the figure.

With the aim of determining the timeline kinetics of neutralizing antibody production in patients with AID, PRNT titers were measured at sequential time points (D0, D3, D4, D5, D6, D7, D14, and D28). The results demonstrated that patients with AID had significantly lower PRNT titers at D5, D14, and D28 than HC. After correction for multiple testing the PRNT titers among AID subgroups showed that they were significantly lower at D28 relative to HC (Figure 3).

**Figure 4:**
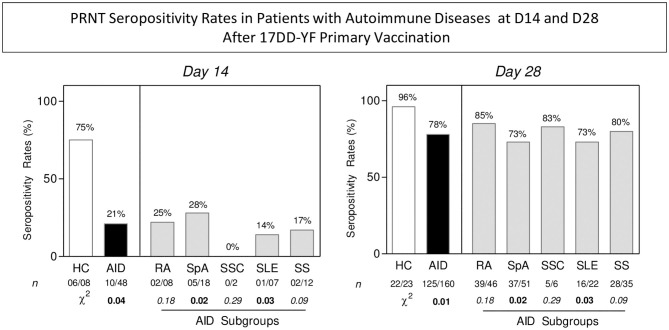
PRNT seropositivity rates in patients with AID at D14 and D28 after 17DD-YF primary vaccination. Levels of 17DD-YF-specific neutralizing antibodies were detected by micro-PRNT, as previously described by Simões et al. (27). Seropositivity rates were calculated with a serum dilution ≥ 1:50 considered the cut-off criterion for PRNT positivity (PRNT ≥ 1:50). The results are presented in bar charts for HC (□), AID (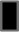), and AID subgroups (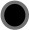). A chi-square test was employed for comparative analysis of PRNT seropositivity rates among groups. A threshold *p* < 0.05 was considered statistically significant. HC, healthy contros; AID, autoimmune patients; RA, rheumatoid arthritis; SpA, spondyloarthitis; SSC, systemic sclerosis; SLE, systemic lupus erythematosus; SS, primary Sjögren's syndrome.

Seropositivity rates at D14 and D28 were further assessed, demonstrating that the seropositivity rate at D14 was significantly lower in patients with AID than those in HC (21 vs. 75%; *p* = 0.04). Comparative analysis among AID subgroups demonstrated overall impaired seropositivity rates at D14 (RA = 25%, SSC = 0%, SS = 17%) with significant differences observed for SpA (28%; *p* = 0.02) and SLE (14%; *p* = 0.03) relative to HC. Seropositivity rates at D28 showed that patients with AID presented late seroconversion profiles, regardless of subgroup, reaching 78% seroconversion relative to D14 (Figure 4).

### Kinetic Timeline of 17DD Viremia

**Figure 5:**
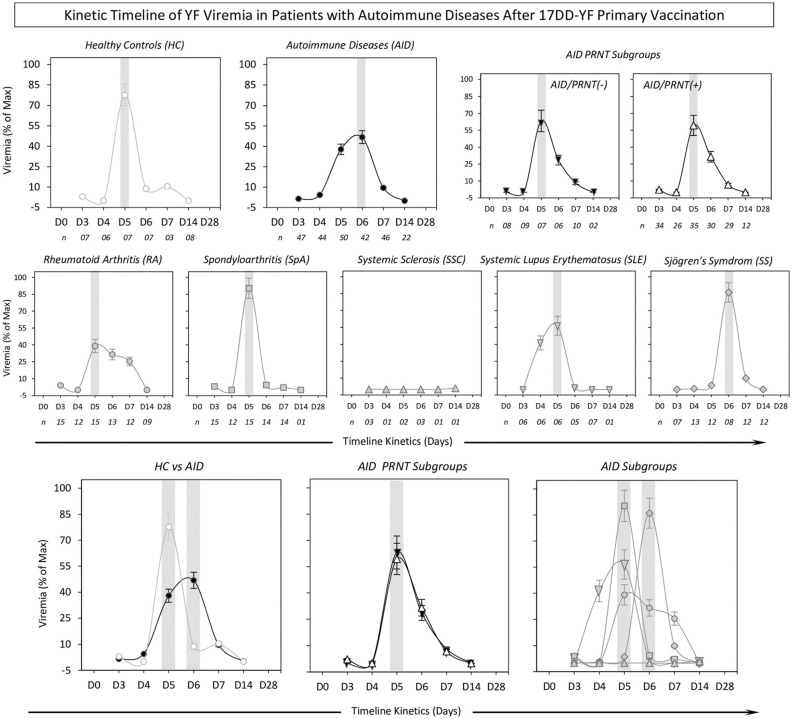
Kinetic timeline of YF viremia in patients with autoimmune diseases after 17DD-YF primary vaccination. Viremia levels (YF viral RNAnemia) were quantified in serum samples by qRT-PCR assay, according to Martins et al. (28). The results are expressed as percentage of maximum viremia levels ± standard error detected for (HC,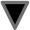; AID,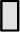; AID–,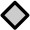; AID+,▵; RA,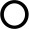; SpA,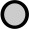; SSC,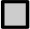; SLE,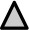; and SS,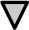) over time after primary vaccination (D3, D4, D5, D6, D7, and D14). The gray background represents the day of the viremia peak. Overlaid kinetic timeline profile of YF viremia is also provided in the figure. The number of samples tested for HC, AID, and AID subgroups is provided in the figure.

Viremia profiles were analyzed at sequential time points (D0, D3, D4, D5, D6, D7, D14, and D28) and the data are presented as the percentage of maximum (Figure 5). Analysis of overall viremia profiles demonstrated that the YF viral RNAnemia peak and global maximum were detected around D5–D6, regardless of AID subgroup. The YF viral RNAnemia peak was slightly later and lower in patients with AID (D6 = 47%) relative to HC (D5 = 78%). Additional analysis was carried out by segregating patients with AID into two subgroups, according to their seroconversion profiles: AID/PRNT(–) and AID/PRNT(+). The day of viremia peak with global maximum values (AID/PRNT(–) = 55%; AID/PRNT(+) = 45%) was detected at D5. Comparative analyses of AID subgroups further demonstrated that global maximum values were detected at around D5 (RA = 39%; SpA = 90%; SLE = 57%) and D6 (SS = 86%). Viremia was undetectable in the SSC subgroup (Table 4).

**Table 4:** Viremia levels in patients with autoimmune diseases after 17DD-YF primary vaccination.

**Groups**	**Viremia peak**	**Viremia level at peak^a^**	***p*-value**
	**(day after vaccine)**	**(Mean copies/mL)**	
**HC** (*n =* 07)	Day 5	8.2 ± 0.7 × 10^3^	
**AID** (*n =* 42)	Day 6	5.9 ± 0.7 × 10^3^	0.16
**AID/PRNT(–)** (*n =* 07)	Day 5	1.3 ± 0.1 × 10^3^	0.18
**AID/PRNT(+)** (*n =* 35)	Day 5	6.3 ± 0.3 × 10^3^	0.61
**RA** (*n =* 15)	Day 5	1.6 × 10^3^	0.17
**SpA** (*n =* 15)	Day 5	11.3 × 10^3^	0.56
**SSC** (*n =* 02)	–	Undetectable	–
**SLE** (*n =* 06)	Day 5	4.8 × 10^3^	0.25
**SS** (*n =* 08)	Day 6	28.2 × 10^3^	0.76

a *Data are reported as mean YF viral copies ± standard error (SE)/mL. Comparative analysis between HC and AID (p = 0.16) and AID/PRNT(–) and AID/PRNT(+) (p = 0.23) were carried out by Mann-Whitney test. ANOVA and multiple comparisons amongst HD and AID subgroups were performed by Kruskal-Wallis (p = 0.20), followed by Dunn's multiple comparison test. HC, healthy controls; AID, autoimmune disease patients; RA, rheumatoid arthritis; SpA, spondyloarthritis; SSC, systemic sclerosis; SLE, systemic lupus erythematosus; SS, primary Sjögren's syndrome*.

## Discussion

This investigation prospectively evaluated AE in response to, and efficacy of, YF primary vaccination in patients with rheumatic AID. Despite data showing that antibody levels were lower than those in controls, consistent seroconversion rates were observed in patients with AID.

A systematic review, including case reports following live vaccinations of immunosuppressed patients, showed that the rate of seroconversion of YF vaccine was high, and better than those of other live vaccines, in patients with AID (34).

Oliveira et al. (35) studied 31 individuals with AID who were inadvertently re-vaccinated. Similar to our results, they reported a seroconversion rate of 87%. Both studies suggest that, although the titers of neutralizing antibodies are lower among patients with rheumatic disease than healthy individuals, they were sufficiently high to confer a protective response (36).

A single study from the Netherlands reported 15 cases of patients with AID (rheumatoid arthritis, pyoderma gangrenosum, and psoriatic arthritis) who received primary YF vaccination, which reported 50% seroconversion (virus neutralization at serum dilution 1:50) in patients using methotrexate (*n* = 8), prednisone (*n* = 1), leflunomide (*n* = 1), and etanercept (*n* = 2) (5). We found a higher of seroconversion rate of 78% than the reported latter study; however, there are some potential reasons for the difference between these studies. First, we included patients who underwent planned vaccination and were under low level immunosuppression and, second, we prospectively evaluated all participants 28 days after vaccination. In the previous study, samples were collected from 15 immune-compromised individuals, vaccinated with the 17DD-YF vaccine between 2004 and 2012, at different times after vaccination (5). The same authors reported that the percentages of early-differentiated memory cells increased over time and concluded that time since vaccination was negatively correlated with the number of specific memory cells (4).

We also evaluated the immune responses in different diseases. As expected, the response in patients with SLE was lower, probably because the disease pathology affects both innate and adaptive immune responses, particularly those of B-cells (36). A diminished response to antigenic challenge in SLE, including vaccinations, has previously been suggested (36, 37). Holvast et al. (37) evaluated 56 patients with quiescent SLE and 18 HC who received influenza vaccination. Fewer patients achieved a titer ≥ 40 to both influenza A strains (75% of patients vs. 100% of controls) (17, 36). Although the humoral response of patients with SLE is decreased, it still fulfills the criteria for influenza vaccine immunogenicity, as agreed upon by the Committee for Proprietary Medicinal Products (38). Therefore, the clinical relevance of such a decreased response remains unclear. Little is known about cell-mediated immune responses to vaccination in patients with SLE, although diminished or disturbed T helper function has been suggested (38). We considered azathioprine ≤ 2 mg/kg/d as low level immunosuppression, and one third of SLE patients were using it in our study, which may have contributed to the low humoral response observed in the SLE group.

Surprisingly, PRNT levels and the seroconversion rate were as low in the SpA group as those observed in SLE. Our hypothesis is that some patients in this group had a history of using biological therapy and that perhaps the washout time was insufficient to reconstitute an immune response (39). Ferreira et al. demonstrated earlier loss of humoral response, triggered by conventional synthetic DMARDs (csDMARDs), combined with biological DMARDs. This was confirmed by the critical decrease in PRNT seropositivity rate to 76%, observed at > 5–9 years post-vaccination in patients with RA receiving combined therapy, in contrast with the standard decline observed in controls and the csDMARD group 10 years after 17DD-YF vaccination (40).

Our study was conducted in patients under low immunosuppression. Antiproliferative drugs, mycophenolate mofetil, calcineurin inhibitors, azathioprine (> 2 mg/kg/day), prednisone (≥ 20 mg/day), methotrexate (> 20 mg/week), or any immunobiological drug were withdrawn for the minimum recommended interval, according to Brazilian guidelines (17).

In the SpA group, 49% were using biological therapies that were withdrawn after the minimum interval, and it is possible this interval (4–5 half-lives) (17) is insufficient to allow reconstitution of immune responses. Future studies of cellular immune signatures, comparing groups receiving different therapies and with various diseases, could help in understanding why patients with SLE and SpA had the lowest antibodies levels.

Previous studies have shown that severe AE are more common in patients with AID, particularly SLE (8). Also, immunosuppressive drugs can increase the risk of AE (9, 10). We did not observe any severe AE; however, we recorded frequent mild AE (34%), which was similar in the control group and to reports from a previous study (41). We did not explore the risks associated with medication, because all patients were under low level immunosuppression.

Our study has some limitations. The number of AID/PRNT(-) is modest and further studies are required to further explore this matter. We did not analyze cellular responses, which could shed some light on the differences in immune responses observed among patients with various diseases. We were unable to analyze medication background, due to sample size restrictions. In addition, we did not include children in this study neither investigate the disease activity on follow-up. We plan to follow patients after 6 and 12 months to study disease activity, and for 5 years to determine cellular and humoral responses over time. Further studies of immunological biomarkers prior and after 17DD-YF primary vaccination would be relevant to add new insights to explain the differences on seroconversion rates observed amongst AID patients according the subgroups of diseases.

In conclusion, our findings support the safety and efficacy of planned primary YF vaccination for patients with AID with low disease activity and receiving low level immunosuppression. These results will help to define target populations and indicators of protection, particularly in endemic countries with high historical rates of YF virus circulation in continuous expansion.

## Data Availability Statement

The datasets generated for this study are available on request to the corresponding author.

## Ethics Statement

The studies involving human participants were reviewed and approved by Ethics Committee of the Hospital Universitário Cassiano Antônio Moraes/EBSERH at UFES. The patients/participants provided their written informed consent to participate in this study.

## Author Contributions

VV, KM, VD, LP-N, AB, and OM-F: designed the research study. VV, OM-F, AT-C, FF, and MM: acquired funding. JD, AC-A, VP-M, IC-R, SL, EM, GT, KO, MB, and SG: conducted experiments. VV, KM, SM, AP, PR, ES, VD, JD, MBG, LS, RD, AG, TZC, BM, FN-B, LR, TBC, EM, MPG, CC, RG, LB, EP, IK, BB, DP, LD, DM, LG, FP, MSG, AB, and FF: field study. VV, KM, SM, AP, PR, ES, TZC, VD, JD, IC-R, and OM-F: acquired data. VV, SM, AP, KM, JD, and OM-F: analyzed data. VV, KM, JD, and OM-F: drafted the manuscript. VV, KM, SM, AP, PR, ES, VD, SG, JD, MBG, LS, RD, AG, TZC, BM, FN, LR, EM, MPG, LP-N, CC, RG, LB, EP, IK, BB, DP, LD, DM, LG, FP, MSG, AB, FF, GP, LM, AC-A, AT-C, VP-M, IC-R, SL, EM, GT, MM, KO, MB, and OM-F: reviewed/wrote the manuscript. All authors contributed to the article and approved the submitted version.

## Conflict of Interest

The authors declare that the research was conducted in the absence of any commercial or financial relationships that could be construed as a potential conflict of interest.
